# The codesign of implementation strategies for children’s growth assessment guidelines in the dental setting

**DOI:** 10.1186/s40900-022-00356-8

**Published:** 2022-05-16

**Authors:** Amy Ruth Villarosa, Della Maneze, Lucie Michelle Ramjan, Ariana Kong, Ajesh George

**Affiliations:** 1 Centre for Oral Health Outcomes and Research Translation (COHORT), Liverpool, 1871 Australia; 2 Western Sydney University, Penrith, 2751 Australia; 3 South Western Sydney Local Health District, Liverpool, 1871 Australia; 4 Ingham Institute for Applied Medical Research, Locked Bag 7103, Liverpool BC, NSW 1871 Australia; 5 Translational Health Research Institute, Western Sydney University, Penrith, 2751 Australia; 6 Faculty of Medicine and Health, School of Dentistry, The University of Sydney, NSW 2006 Camperdown, Australia

**Keywords:** Codesign, Implementation, Childhood, Overweight, Obesity, Dental

## Abstract

**Background:**

Considering the interdisciplinary role dental staff can play in addressing overweight and obesity in childhood, this study aimed to codesign guideline implementation strategies for children’s growth assessment and dietary advice guidelines in the dental setting.

**Methods:**

This qualitative study utilised principles of codesign and appreciative inquiry through a series of four, two-hour focus groups with dental staff and parents. Focus groups were analysed using content analysis.

**Results:**

Discussion fell into two main themes, engaging patients throughout their care journey and supporting staff to engage with the guidelines. Six strategies were developed within these themes: (1) providing growth assessment information to patients and families before appointments, (2) providing refresher training to staff, (3) involving dental assistants in the growth assessment, (4) keeping dental staff updated regarding referral outcomes, (5) culturally appropriate information resources for patients and families, and (6) enabling longitudinal growth tracking in patient information systems.

**Conclusions:**

This study successfully designed six implementation strategies for children’s growth assessment guidelines in the dental setting. Further research is required to determine their impact on guideline adherence.

**Supplementary Information:**

The online version contains supplementary material available at 10.1186/s40900-022-00356-8.

## Plain English summary

Being above a healthy weight in childhood is a major public health issue. In parts of Australia, dental staff need to screen for and promote healthy weight among children. As this is not a normal part of dental care, it could be hard for this change to come about. So, this study aimed to create strategies to help dental staff to screen for and promote healthy weight among children. As we wanted those impacted by the strategies to have a say, we worked with public dental staff and parents. When health staff and the community come together to design ways to improve health care, this is known as codesign. In groups, these people codesigned a series of strategies. Strategies for parents included: (1) informing parents about what to expect in their child’s appointment; and (2) creating resources for parents from other cultures. Strategies for dental staff included (1) ensuring staff were trained; (2) involving the whole dental team to save time; (3) ensuring dental staff heard back from services they sent children to for healthy weight support; and (4) creating a way to record children’s growth over time. Bringing in both parents and dental staff gave them a voice to codesign strategies to help dental staff screen and promote healthy weight among children. This produced a suite of strategies that were appropriate for all involved.

**Supplementary Information:**

The online version contains supplementary material available at 10.1186/s40900-022-00356-8.

## Introduction

Overweight and obesity among children is a serious public health concern around the world. Between 1980 and 2013, the global prevalence of overweight and obesity among children and adolescents increased by almost 50%, with more than a fifth of children in developed countries classified as experiencing overweight or obesity [1]. This is of concern, as children who experience overweight or obesity are more likely to continue to experience overweight or obesity in adulthood, develop noncommunicable diseases [2], and experience low self-esteem, depression, poor quality of life, bullying and social isolation [3, 4]. Managing overweight and obesity in childhood has therefore been an ongoing focus of both national and global health strategies [4]. Although many health promotion and disease prevention interventions have been developed to reduce overweight and obesity among children [4], the prevalence of childhood obesity remains high [1].

In response to this, the Australian National Health and Medical Research Council (NHMRC) developed clinical practice guidelines recommending that growth of children be monitored using US-CDC or WHO Body Mass Index (BMI) percentile for children aged 2–18 years, and the WHO charts for children aged younger than two years [5]. These guidelines recognise general practitioners (GPs) or practice nurses as the health care providers responsible for these practices [5]. However, research suggests that weight assessment and promotion is often not prioritised in general practice, with over 92% of GPs unaware of the NHMRC clinical practice guidelines [6], and less than 5% of GPs routinely checking children’s weight [7]. There is potential for other health practitioners to address overweight and obesity in children, including dental practitioners [8]. In fact, dental practitioners are well placed for this, as they often see children on a regular basis for periodic oral examinations and could conduct serial growth assessments [8]. Furthermore it is known that being below or above a healthy weight can share risk factors with tooth decay, such as nutrient poor diets, or excess consumption of sugar and sugar-sweetened beverages, and thus identification of these behaviours is already of relevance to dental practice [9–11].

In light of the interdisciplinary role that dental practitioners can play in children’s healthy weight promotion, Australia’s most populated state, New South Wales (NSW), released guidelines for the conduct of children’s growth assessments in public oral health services [12]. However, research highlights that the success of clinical guideline implementation is variable [13]. When implementing children’s growth assessment guidelines in the dental setting, studies have cited lack of training, fear of appearing judgemental, fear of patient rejection, and the complexity of providing healthy weight counselling in a sensitive way, as significant barriers to compliance [10]. Thus there is a growing need to provide strategies to address these factors and optimise the uptake of new clinical guidelines into practice [14]. It is anticipated that guideline implementation strategies will be required to implement children’s growth assessment guidelines in the dental setting, which can include the provision of education and training, equipment and resources, as well as various incentives [15].

Yet to date, no published research explores the development of implementation strategies for this purpose, nor used co-design as the approach. Co-design is a participatory approach that brings together patient and staff experiences with inputs from other stakeholders to improve health service delivery [16]. It acknowledges a clear link between staff and patient experiences in the delivery of healthcare, and solutions designed in this way are more likely to be feasible and sustainable, as they are acceptable to all stakeholders involved [16].

## Methods

### Aims

The aim of this study was to design strategies that would facilitate the implementation of children’s growth assessment and dietary advice guidelines in the dental setting.

### Study design

This study followed a qualitative, appreciative inquiry approach. Appreciative inquiry allows a shift from the traditional research approach of identifying problems in a system, to an approach of identifying what can be done to improve the system [17]. This can promote organisational change, moving away from a potential culture of blame towards self-determined change, which is particularly useful when implementing guidelines such as children’s growth assessment guidelines in the dental setting [17]. This approach allows different members within an organisation to engage in their own research to improve service delivery, and aligns with the principles of codesign, which aims to engage staff and end users in the development of solutions for healthcare delivery [16, 17]. These approaches were thus incorporated to enable the development, feasible, sustainable strategies that would be acceptable by all individuals involved in the guidelines, both staff and end users. All results in this study were presented according to the COREQ reporting guidelines [18]. This study was a single phase of a multi-phase, sequential mixed methods project aiming to develop and evaluate the effectiveness of guideline implementation strategies for children’s growth assessment guidelines in the dental setting.

### Theoretical framework

The integrative model of behavioural prediction (IM) underpinned this study (Additional file 1), which assumes that intentions are the immediate antecedents of behaviour, with environmental factors, skills and awareness as moderators to this relationship [19]. Intentions, in turn, are considered a function of attitudes, perceived normative pressure and self-efficacy [19]. All study findings were framed around the IM.

### Context

This study was undertaken across two Local Health Districts in Greater Western Sydney, NSW, Australia between July to August of 2020. One district (D1) was larger, with a population around 1 million people served by nine public dental clinics. In this district, under half (46%) of people spoke English at home, and regions within this district experienced anywhere from the 1st to the 10th decile of the Index of Relative Socio-economic Disadvantage (IRSD) [20, 21]. This indicated this was a diverse community, with individuals from the 10% most to the 10% least disadvantaged individuals in the country. The other district (D2) was smaller, with a population of almost 350,000 people served by three public dental clinics. Most people (82%) within this district spoke English at home, and regions within this district experienced anywhere from the 2nd to the 9th decile of the Index of Relative Socio-economic Disadvantage, however most were above the 7th decile, indicating this population experienced less disadvantage than D1 [20, 21].

### Participants and sampling

In line with the principles of co-design, recruitment strategies endeavoured to sample both the staff that would be engaging in the new guidelines, as well as parents who would be end-users of these guidelines and could provide consumer insight. Purposive sampling was used to ensure a spread of dental therapists (DTs), oral health therapists (OHTs) and dental assistants (DAs) employed at the 12 participating public dental clinics were recruited. DTs and OHTs are the main practitioners that treat children in NSW public dental services, and thus would be the main staff involved in the new guidelines. DAs are partnered with DTs and OHTs to aid them throughout the appointment, and therefore could also play a role. Focus group times were organised during free time slots in clinic appointment books. Eligibility criteria for dental staff can be seen in Table 1. Invitation flyers, information sheets and consent forms were circulated to eligible staff via email.

**Table 1:** Eligibility criteria for participants

Dental staff criteria	Parent criteria
Is a practising dental therapist, oral health therapist or dental assistant Works in public dental services within either of the participating districts	Has at least one child aged 2–18 years Lives within either of the participating districts

Parents (P) of children living within either of the participating districts were also recruited using purposive sampling. This sampling technique was used in an attempt to obtain diversity in parents regarding cultural and linguistic diversity, socioeconomic status, level of education and gender. Refer to Table 1 for eligibility criteria for parents. Word of mouth recruitment and snowballing was used, where dental staff and researchers invited eligible parents to contact the principal investigator, who in turn provided further information, consent forms and scheduled them into a focus group time slot. Invited participants included attendees at the public dental services, parents known to the research team, and parents accessing a multicultural health service.

### Data collection

A total of four focus groups were conducted, two at each participating district. These focus groups were conducted virtually over Zoom and lasted approximately two hours, with only researchers and participants present. Focus groups 1, 3 and 4 were facilitated by AV and AG, and focus group 2 also had LR and DM present. All facilitators were experienced qualitative researchers, with the lead investigator being formally trained in appreciative inquiry. The lead investigator provided all other facilitators with training regarding appreciative inquiry and the intended format of the focus groups. At the commencement of each focus group, while dental staff and parents were together, a facilitator gave an introduction explaining the guidelines and codesign process in simple terms, and emphasising the desire for all participants to have a voice based on their firsthand experiences. After initial introduction, dental staff and parents were moved into separate breakout rooms to ensure participants were comfortable and able to express themselves freely, particularly as dental staff may have a position of power in respect to the parents. In these separate rooms they recalled and share positive experiences with either providing or receiving health promotion and identify themes that they perceived led to the success of those experiences. Each room was asked to choose the three most important themes that could be useful in the implementation of the children’s growth assessment guidelines. These themes were written down by facilitators, with the list verified by all participants.

Following this, the breakout rooms were combined into a single room, where participants identified the top four themes overall. This involved either combining common themes between groups or eliminating less relevant themes. At this stage, facilitators used the written list of themes to ensure themes were objectively and accurately presented, ensured all participant voices were sufficiently heard, and avoided leading questions to ensure the process was led by participants. Due to the appreciative inquiry approach, only positive factors were inherently being discussed, and as a result there were very few points of disagreement, and minimal issues in bringing together the staff and parents. Where any disagreement arose, facilitators enabled all participants to express and justify their opinions, and in all cases, the act of doing this enabled others to gain perspective and for consensus to be achieved. Participants were then prompted to use the final themes to co-design actionable implementation strategies that could be used with the growth assessment guidelines. Toward the end of each workshop, strategies proposed by participants were written by facilitators onto a screen shared document. Facilitators ensured participants verified these strategies at this time, and where required, modifications were made. One focus group (focus group 4) had only dental staff participate, and as a result they engaged in a single continuous focus group where all the above steps were still undertaken, aside from sharing and combining themes with the other group. All focus groups were recorded, and investigators also took field notes, some of which were verified by participants.

### Data analysis

All recorded focus groups were professionally transcribed verbatim and reviewed by a study investigator (AV) for accuracy. All participants were assigned pseudonyms for confidentiality (see Additional file 2 for all pseudonyms with basic participant characteristics), after which, content analysis was undertaken. All study investigators familiarised themselves with some of the focus group transcripts, with AV and AK reading all focus group transcripts. Transcripts were initially analysed to develop preliminary coding frames, with two preliminary coding frames independently developed, one by AV, and the other by DM and AK. A consensus meeting with the entire study team was held to review both coding frames, negotiate divergent viewpoints and decide upon the best structure for the final coding frame. Following this the final coding frame was piloted and refined by AV, and a final consensus was achieved with the study team. The final coding frame included a hybrid of both concept-driven categories (deductive) derived from the theoretical framework adopted for the study, and data-driven categories (inductive) generated by participants’ themes. All relevant study data were categorised into this final coding frame.

### Research team and reflexivity

All members of the research team completed a reflexivity questionnaire following data collection to allow researchers to reflect on how their backgrounds, beliefs and prior relationships may have impacted data collection. See Additional file 3 for the full reflexivity questionnaire. All investigators had experience in facilitating and analysing focus groups, with one researcher (LR) having an extensive background in qualitative research. Furthermore, all researchers had a health-related professional background, which allowed unique insight into the research area and in-depth probing of participants. Most investigators shared the beliefs that overweight in childhood, as well as expanding the scope of clinical practice were challenging and sensitive topics to address, however acknowledged that a strength-based approach could be an effective way to conduct the study. Only one researcher (DM) had close relationships with some of the participants, with one participant being a niece, and the other being a friend, however upon reflection it was not believed to impact on the data collected. To address any bias caused by this relationship, it was ensured that this investigator did not facilitate the focus group alone and played more of a scribe role than facilitator. Other researchers noted that some participants were current or prior colleagues (AG and AV), however considering the topics being discussed it was anticipated this would not significantly impact data collection. Further details on each researcher can be seen in Additional file 4.

### Rigour and trustworthiness

Throughout the conduct of this study, various strategies were used to ensure the rigour and trustworthiness of study findings, and specifically to address credibility, transferability, confirmability and dependability [22]. The credibility of research findings was ensured by prompting participants to generate initial themes on which the coding frame was based, through the use of multiple independent researchers when undertaking analysis and through data triangulation derived from different perspectives. These included perspectives from dental healthcare workers as providers of health promotion and parents as consumers of health promotion messages [22]. This data triangulation, along with recording of focus groups and field notes from researchers, also helped ensure confirmability of results [22]. Dependability was also ensured through the recording of focus groups, discussions and careful documentation of the steps undertaken in the research processes [22]. Finally, transferability was maximised through the use of thick description to sufficiently detail the context in which data was collected, as well as involvement of different local health districts, different types of dental staff, and parents from a range of cultural backgrounds and educational levels [22].

## Results

### Demographics

A total of 20 dental staff participated in the four focus groups and eight parents participated across three focus groups. Of the dental staff, five were dental assistants and 15 were dental therapists and most reported receiving training regarding growth assessment. All eight parents who participated in the study were females with age ranging from 29 to 54 years (mean 40.25 years). Full demographic characteristics of participants are outlined in Table 2.

**Table 2:** Demographic characteristics of participants

Characteristic	*n* (%)
*Dental staff *(*n* = *20*)	
Current position	
Dental Assistant	5 (25.0)
Dental Therapist	8 (40.0)
Oral Health Therapist	7 (35.0)
*Sex*	
Male	1 (5.0)
Female	19 (95.0)
Age (mean ± SD)	42.75 ± 12.14
*Highest level of education*	
Diploma/Certificate	10 (50.0)
Bachelor	7 (35.0)
Graduate Certificate/Diploma	3 (15.0)
*Received training regarding growth assessment for children*	
Yes	18 (90.0)
No	2 (10.0)
*Parents *(*n* = *9*)	
Sex	
Male	0 (0.0)
Female	9 (100.0)
Age (mean ± SD)	38.33 ± 9.24
Number of Children (median, range)	2, 2–5
Age of Youngest/Only Child (mean ± SD)	6.44 ± 4.32
Age of Oldest Child (mean ± SD)^†^	12.78 ± 8.48
*Highest level of education*	
Diploma/Certificate	3 (33.3)
Bachelor	3 (33.3)
Masters	3 (33.3)
*Children have received a growth assessment in the dental setting*	
Yes	4 (44.4)
No	5 (55.6)

### Content analysis

Content analysis resulted in the development of a coding frame which was constructed to align with the IM framework and consisted of two major themes: (1) engaging patients throughout their care journey; and (2) supporting staff to engage with the guidelines. This frame also had six subthemes, three under each major theme. All themes and subthemes were discussed by all four focus groups. The perspectives of the patients as seen in the first theme of this coding frame were vital background factors that could shape stereotypes and stigmas related to the guidelines. As per the IM, these factors influence intentions and environmental constraints when engaging in the guidelines. Please refer to Additional file 5 for additional quotes for each theme and subtheme.

## Theme 1: Engaging patients throughout their care journey

Across all four focus groups, parents and dental staff alike highlighted the importance of keeping patients and their families engaged throughout their entire care journey, from before the appointment through to after the appointment. This was considered key if implementation of the children’s growth assessment guidelines was to be successful.

### Setting expectations before the appointment—“it’s not a surprise”

Parents and dental staff from all four focus groups discussed the importance of patients and their families being aware of these guidelines prior to attending their appointment. This would ensure patients and their families already knew what to expect coming to their appointment and would not feel as though they were being judged when they were invited to participate in a growth assessment.

“…*if someone's coming for an appointment, telling them beforehand that this is going to happen, so they don't feel like they've walked in the front door and you looked at their child and went, wow, that child looks overweight I'm going to do a height and weight on them*.” (Sarah – P, D1)

Within two focus groups, there were some comments from dental staff and parents that prior information about the guidelines be particularly beneficial to prepare teenagers and children with special needs who may be more sensitive or apprehensive about receiving a growth assessment.

When it came to specific strategies, participants from all focus groups suggested incorporating this information into the existing appointment booking processes. In NSW, initial appointments for public dental consultations are generally booked through a central call centre, following which a reminder letter will be sent to patients. Thus, parents and dental staff discussed the potential for some brief information regarding the growth assessment to be provided either through the call centre or with reminder letters.

“…*they could tell us when we book an appointment or even you know how you get a letter sent out in the mail with the appointment time and stuff on it, could they add it into that*” (Joanne – P, D2)

### Personalised care during the appointment—“tailored messaging… increases engagement”

Parents and dental staff from all focus groups highlighted the need for personalised care during the appointment, and how this was vital when conveying growth assessment information to patients and their families. When receiving health information, parents and dental staff from all three focus groups agreed that information tailored to the specific experiences of each patient, that could be understood by both the child and parent, resulted in the best outcomes. One parent summarised that they preferred “*not being talked to by a health professional with one narrow message. The tailored messaging to the child, plus the parents I think increases engagement*.” (Mai – P, D1).

However, one area in which personalised care was limited was regarding cultural sensitivity. Parents from culturally and linguistically diverse (CALD) backgrounds in one focus group emphasised the need to be culturally sensitive when engaging with these guidelines to avoid feelings of being judged or criticised. One parent reflected on her own experience:

*“I felt all the time that criticism, even in the way they were speaking to me, they spoke like … [by] raising their voice [I was going to] understand better and it was not the case. I could understand, but I couldn’t speak. That was my problem*.” (Isabella – P, D1)

Dental staff from three focus groups agreed that acknowledging various cultural backgrounds was an important part of personalised care and proposed that more educational resources developed for people from culturally and linguistically diverse backgrounds would be helpful. One dental staff member stated, “*it's very, very important if you're going to have resources [there] has to be a translation*” (Lucy – OHT, D1). However, it was acknowledged that some districts were more multicultural than others, and thus the need for multicultural resources varied.

### Continuity of care after the appointment—“I knew where… we are headed”

Parents from all three focus groups discussed that they wanted continuity of care when being referred to weight management services, so they could have a seamless transition while still being followed up at subsequent dental appointments.

“…*some sort of clear follow through plan so that we could always go back to someone and get reassured, rather than it being this one event experience for the parent and child, and then they're move on to be referred to all these other programs, and it's never discussed again*.” (Mai - P, D1)

Dental staff from all focus groups also appreciated the benefits of being able to follow through with patients. However, in two focus groups, dental staff also highlighted that following up patients after referral to other services was sometimes difficult as these services did not always send updates or discharge summaries back to the referral clinic.

“*I would never really know if they’ve even done the Go4Fun [children’s healthy weight program] or anything like that unless I ask them the following time if they have or have not or whatever*.” (Rosalie - OHT, D2)

Dental staff suggested the routine provision of such updates could be a strategy to ensure that they remain informed about their patients’ progress and allowed for follow up.

## Theme 2: Supporting staff to engage with the guidelines

Focus group participants also acknowledged the support dental staff required to engage with the children’s growth assessment guidelines. This support was needed to increase intentions to adhere to guidelines, improve awareness and skills, and address environmental constraints.

### Increasing intentions to engage in guidelines—“this is a new practice that’s come into play”

Most dental staff across all focus groups demonstrated positive attitudes and believed the children’s growth assessment guidelines were beneficial. Specifically, they emphasised these guidelines could help provide assessments to children that do not regularly access other health services. Furthermore, dental staff from three out of the four focus groups, and parents from all three focus groups expressed that although a sensitive topic, especially with teenagers, most parents would accept the growth assessment guidelines and understood that this had a connection to oral health. Therefore, staff displayed a good amount of confidence with adhering to the guidelines once they explained to the patient that this was part of standard care across all medical services.

“…*you just say to them that this is a new practice that’s come into play with all of health. You’ll be asked at doctors – you know, various different medical appointments and it’s just part of the normal procedure. Are you happy to go ahead with it? I don’t think I’ve ever had someone say no to me by saying it that way*.” (Fatima – OHT, D2)

However, one staff member was concerned about the appropriateness of growth assessment for children and thought it would not be accepted by parents. As a result, this staff member did not display high self-efficacy in engaging with the guidelines. “*I understand why the government wants to do this, but I do feel very cornered in giving people information that they most probably don’t want to know*.” (Leslie – DT, D2).

Regarding specific strategies to feel more confident in adhering to the guidelines, staff proposed that there should be more informational pamphlets available to provide parents a rationale for the growth assessments.

### Improving awareness and skills—“you’ve got to give [them] the knowledge and the training”

Dental staff from all four focus groups and parents from two out of three focus groups thought it was vital for staff to be adequately trained regarding the growth assessment guidelines. Staff from three focus groups discussed that training would help ensure consistent messaging throughout the dental service. Furthermore, one staff member highlighted that although some staff had received training, they still lacked sufficient skill when performing the growth assessments.

To address this, staff from three focus groups proposed future training programs, which should have more in depth information about the referral pathways that were in place. Staff from two focus groups discussed the potential for direct collaboration with other allied health staff to improve their skills in the care of their patients:

*“A dietitian point of view is different from a dental therapist, oral health therapist point of view... I think it's a collaboration is what we need, and we need to be trained differently.”* (Marie – DT, D1)

### Addressing environmental constraints—“we haven’t got enough time”

Dental staff from all focus groups explained that environmental factors, things beyond their control in their work environment and context, could impact engagement of both them and their patients in the growth assessment guidelines. Time limitations were discussed in all focus groups, as dental therapists have limited time to complete consultations. Staff proposed that training dental assistants to conduct the growth assessment could be a potential strategy to save time by enabling dental therapists to simultaneously conduct other activities. One dental therapist explained, “*the only criticism I’ve got of it is that we haven’t got enough time and if we can train the assistants to be able to take the measurements, that’s a really big help*.” (Jane – DT, D1).

Staff from three focus groups and parents from one focus group also discussed the location of the scales and timing of the growth assessment as a factor that could influence the outcomes of growth assessments. With some scales in central locations and others in private rooms, and some appointments being a less appropriate time for growth assessments, such as emergency or acute consultations, staff wanted the freedom to decide when and where these assessments were conducted. They highlighted this could help encourage the cooperation of patients and families.

*“Whether to do it at the first appointment or whether it is … more appropriate to make sure that we look after the pain rather than concentrate on height and weight at the beginning…”* (Bianca – DA, D1)

Dental staff from two focus groups raised issues with how productivity was measured in their clinics. Performing dental treatments such as fillings could credit staff with higher productivity for the same amount of time required when compared to health promotion activities. They therefore explained that a system which better recognised the time required for health promotion would further incentivise implementation of the growth assessment guidelines.

“…*if … you’ve done three fillings in one side that’s more productive than what the dental health education is or the diet discussion in their eyes and in their view*.” (Leslie – DT, D2)

Finally, staff from one focus group thought the patient information systems they used should include a longitudinal growth chart. Staff clarified that the existing system catalogued a new chart for each new growth assessment, rather than plotting them longitudinally. They emphasised that a longitudinal growth chart would enable them to better monitor children over time.

“…*show them the changes because some kids belong at the 80th percentile, some kids belong at the 20th percentile... I would like to be able to compare over visits much more easily than we can*.” (Peter – DT, D2)

### The designed strategies

**Fig. 1:**
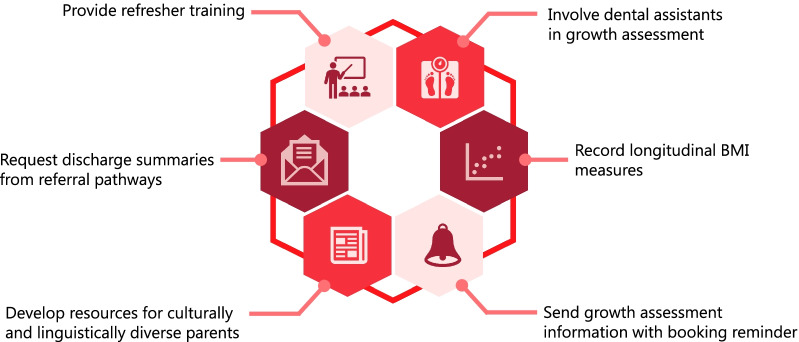
Strategies codesigned by participants

From the above themes, six implementation strategies were designed by participants. For dental staff, these included: (1) the use of refresher training to ensure staff were trained on a regular basis; (2) the involvement of dental assistants in the growth assessment to save time for dental therapists to engage in other tasks; and (3) developing a way to record and present children’s growth measurements across time so that staff could track a child’s progression. For parents, implementation strategies involved: (1) the inclusion of growth assessment information in the booking reminder letters they received prior to attending appointments, (2) developing information resources for parents from CALD backgrounds; and (3) requesting referral pathways to send discharge summaries back to the referring dental clinics. See Fig. 1 for all strategies.

## Discussion

This qualitative study aimed to use the principles of appreciative inquiry to codesign implementation strategies for children’s growth assessment and dietary advice guidelines in the dental setting. Strategies proposed in this study were based around two main themes: (1) engaging patients throughout their care journey, and (2) supporting staff to engage with the guidelines. Within the theme of engaging patients, participants proposed strategies to provide information regarding the guidelines prior to the appointment, provide tailored and culturally appropriate information during the appointment, and to ensure continuous care and sufficient follow-up after the appointment.

This theme of engaging patients is consistent with the existing literature, which highlights the importance of accounting for the needs of patients in implementation [23]. Specifically, studies in other healthcare settings have highlighted the importance of setting patient expectations through provision of information, being correlated with patient satisfaction [24]. In addition, personalised care, specifically regarding the information patients receive and cultural sensitivity, is also known to be vital in guideline implementation [25, 26]. Furthermore, research has found provider continuity to be associated with improved patient outcomes and satisfaction [27].

The second theme of supporting clinicians included strategies to provide educational resources and training, involve dental assistants to save time, and improve location of scales, timing of growth assessments, productivity appraisal and documentation of longitudinal growth assessments. Other studies also found that when it came to children’s healthy weight and sugar consumption, dental staff were in need of educational materials, especially for nutrition-related advice, and further training [10]. Provision of education is also an effective implementation strategy for dental staff [28]. Research also highlights the need for innovative solutions to time constraints, which are a known challenge for childhood obesity interventions in the dental setting, and the importance of good productivity systems for staff, with pay for performance an effective implementation strategy for dental staff [28, 29]. Furthermore, current practice guidelines emphasise the importance of the location of scales and timing of growth assessment for privacy purposes, and the use of longitudinal growth charts for children’s growth assessments [5, 30].

Although some dental research has based implementation strategies on qualitative data and feedback from users [31], to our knowledge there are no existing studies that use the principles of codesign for implementation strategies in the dental setting. Furthermore, to date, no published research explores the development of implementation strategies for children’s growth assessment and dietary advice in the dental setting. Thus, this study provides rich insight into a unique approach for developing implementation strategies. Although there is a lack of empirical evidence regarding codesign approaches, it is known that codesign can be beneficial for practitioners and research outcomes, not only producing materials with increased relevance and acceptability for end users, but also resulting in a sense of support and enthusiasm for the intervention [32]. In light of the mounting evidence surrounding the links between oral health and general health, and thus the ever-expanding interdisciplinary role of dental staff [8, 33], research on the use of codesign in this context is much needed to inform such public health initiatives.

Despite this study being the first of its kind in this area, there are some limitations that should be acknowledged in the interpretation of the data. Firstly, the majority of participants in this study were female, and no male parents participated. However, 87.9% to 98% of the dental therapist, oral health therapist and dental assistant workforces in Australia are female, thus the study sample can be seen as representative of these figures [34–36]. In addition, Australian data indicates that not only are female parents more likely to be unemployed, but employed female parents more often utilise work arrangements that enable them to care for their children during the day [37]. This would significantly impact the demographic characteristics of parents available during business hours for this study. Nonetheless, the lack of male parent perspectives in this study could impact representation of single parent, and other households with only male parents, and therefore results should be interpreted with caution. Furthermore, the education level of parents was higher than average for Australia, with all parents holding an educational qualification beyond high school, compared to around 70% of Australians of the same average age [38]. Although it is anticipated these strategies would remain highly applicable to parents with other educational levels, this difference should still be considered when applying proposed strategies to other populations. This study was also limited to two urban districts in greater western Sydney, and although these districts have variability in cultural diversity and socioeconomic status, proposed strategies were based on the needs of individuals within these districts, and thus may be only applicable for districts with similar needs. Furthermore, due to the word of mouth and snowballing recruitment techniques, the exact number of individuals approached for the study was not documented, thus response rates could not be determined. Finally, while focus groups were conducted virtually for this study due to COVID-19 restrictions, research has highlighted the value of online platforms for qualitative research, with video conferencing methods having the potential to produce data of similar quality to face-to-face methods [39]. All participants were able to use and access Zoom, and most were already familiar with the platform. Detailed instructions were provided to all participants, and telephone numbers were also collected as a contingency plan in the case of technical difficulties, however there were minimal technical issues experienced during the focus groups. Thus it is anticipated that there is negligible impact on data collected using this method.

Despite these limitations, this study has provided unique insights from key players and produced novel data regarding the codesign of implementation strategies in the dental setting. These findings could have a significant impact on how the public health concern of overweight and obesity in childhood is addressed in the dental setting. Further research could involve process evaluations to explore the experience of participants during similar codesign approaches. Future phases of this research project will involve quantitative evaluations to determine the effectiveness of co-designed strategies in improving guideline adherence.

## Conclusions

This study used a collaborative, co-design approach to create potential implementation strategies for children’s growth assessment guidelines in the dental setting. These strategies were focused around engaging patients and supporting staff so that both parties could adhere to the guidelines. This collaborative approach has provided a voice for key players in the design of effective, sustainable, and feasible implementation strategies for guidelines to assess children’s growth in the dental setting. This will ultimately assist in addressing the public health concern of overweight and obesity in childhood.

## Supplementary Information

**Additional file 1**. The Integrative Model of Behavioural Prediction asapplied to the study.

**Additional file 2**. Focus group case classification table.

**Additional file 3**. Focus group reflexivity questionnaire.

**Additional file 4**. Characteristics of the research team.

**Additional file 5**. Table of additional quotes.

## Data Availability

The datasets used and/or analysed during the current study are available from the corresponding author on reasonable request.
