# Supplementary material for: The codesign of implementation strategies for children’s growth assessment guidelines in the dental setting
Source: Res Involv Engagem. 2022 May 16;8:19. doi: 10.1186/s40900-022-00356-8 (PMC9109434; doi:10.1186/s40900-022-00356-8)
Supplement: Supplementary file 1 — Additional file 1. The Integrative Model of Behavioural Prediction asapplied to the study. [file 40900_2022_356_MOESM1_ESM.pptx]

## Slide 1
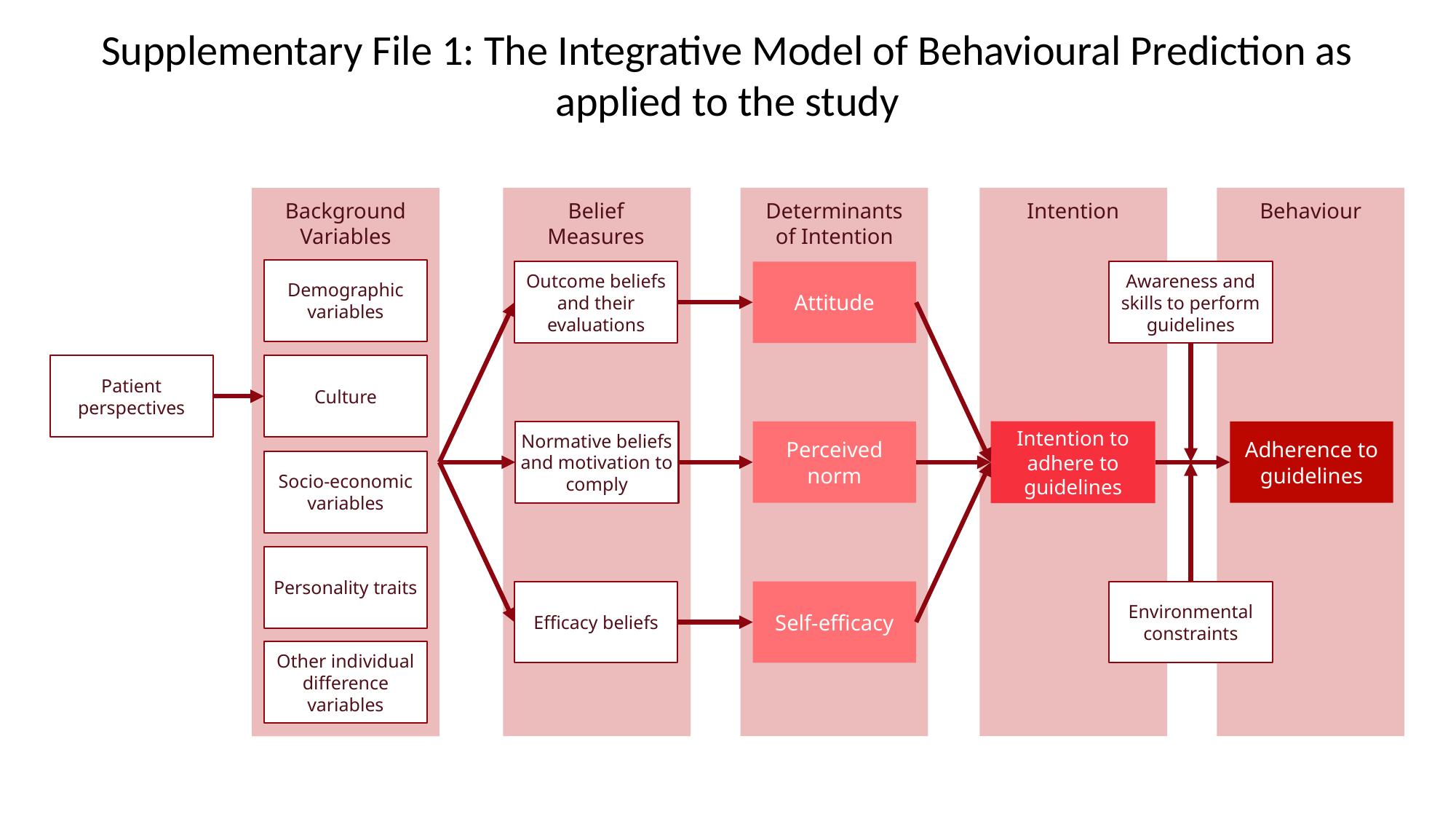

Supplementary File 1: The Integrative Model of Behavioural Prediction as applied to the study
Background Variables
Belief Measures
Determinants of Intention
Intention
Behaviour
Demographic variables
Outcome beliefs and their evaluations
Attitude
Awareness and skills to perform guidelines
Culture
Intention to adhere to guidelines
Normative beliefs and motivation to comply
Perceived norm
Adherence to guidelines
Socio-economic variables
Personality traits
Environmental constraints
Efficacy beliefs
Self-efficacy
Other individual difference variables
Patient perspectives
