# Supplementary material for: The codesign of implementation strategies for children’s growth assessment guidelines in the dental setting
Source: Res Involv Engagem. 2022 May 16;8:19. doi: 10.1186/s40900-022-00356-8 (PMC9109434; doi:10.1186/s40900-022-00356-8)
Supplement: Supplementary file 2 — Additional file 2. Focus group case classification table. [file 40900_2022_356_MOESM2_ESM.docx]

# Supplementary File 2: Focus group case classification table

| **Pseudonym** | **Participant type** | **Gender** | **District** | **Focus Group** |
| --- | --- | --- | --- | --- |
| Marie | Dental Therapist | Female | District 1 | Focus Group 1 |
| Lucy | Oral Health Therapist | Female | District 1 | Focus Group 1 |
| Sofia | Dental Therapist | Female | District 1 | Focus Group 1 |
| Ayla | Dental Therapist | Female | District 1 | Focus Group 1 |
| Sarah | Parent | Female | District 1 | Focus Group 1 |
| Mai | Parent | Female | District 1 | Focus Group 1 |
| Amita | Parent | Female | District 1 | Focus Group 1 |
| Natasha | Dental Assistant | Female | District 1 | Focus Group 1 |
| Bianca | Dental Assistant | Female | District 1 | Focus Group 2 |
| Dhara | Dental Assistant | Female | District 1 | Focus Group 2 |
| Jane | Dental Therapist | Female | District 1 | Focus Group 2 |
| Maryam | Dental Therapist | Female | District 1 | Focus Group 2 |
| Ashanti | Oral Health Therapist | Female | District 1 | Focus Group 2 |
| Isabella | Parent | Female | District 1 | Focus Group 2 |
| Ximena | Parent | Female | District 1 | Focus Group 2 |
| Martina | Parent | Female | District 1 | Focus Group 2 |
| Natalie | Dental Assistant | Female | District 2 | Focus Group 3 |
| Hannah | Dental Assistant | Female | District 2 | Focus Group 3 |
| Fatima | Oral Health Therapist | Female | District 2 | Focus Group 3 |
| Leslie | Dental Therapist | Female | District 2 | Focus Group 3 |
| Joanne | Parent | Female | District 2 | Focus Group 3 |
| Lisa | Parent | Female | District 2 | Focus Group 3 |
| Emma | Oral Health Therapist | Female | District 2 | Focus Group 4 |
| Peter | Dental Therapist | Male | District 2 | Focus Group 4 |
| Melissa | Dental Therapist | Female | District 2 | Focus Group 4 |
| Rosalie | Oral Health Therapist | Female | District 2 | Focus Group 4 |
| Penelope | Oral Health Therapist | Female | District 2 | Focus Group 4 |
| Anna | Oral Health Therapist | Female | District 2 | Focus Group 4 |
