# Supplementary material for: The codesign of implementation strategies for children’s growth assessment guidelines in the dental setting
Source: Res Involv Engagem. 2022 May 16;8:19. doi: 10.1186/s40900-022-00356-8 (PMC9109434; doi:10.1186/s40900-022-00356-8)
Supplement: Supplementary file 3 — Additional file 3. Focus group reflexivity questionnaire. [file 40900_2022_356_MOESM3_ESM.docx]

Focus group reflexivity questionnaire

As a facilitator of one or more focus groups, it is important to reflect about how you may have influenced the data collected. The following questions relate to your experience(s) when facilitating focus groups.

Q1 Investigator name: _________________________________

Q2 Did you have any prior relationships with participants?

- Yes (1)
- No (2)

Display This Question:

If Did you have any prior relationships with participants? = Yes

Q3 Please describe these relationships

________________________________________________________________

________________________________________________________________

________________________________________________________________

________________________________________________________________

________________________________________________________________

Display This Question:

If Did you have any prior relationships with participants? = Yes

Q4 Please reflect on how these relationships may have impacted data collection

________________________________________________________________

________________________________________________________________

________________________________________________________________

________________________________________________________________

________________________________________________________________

Q5 Describe your beliefs and values, including cultural and theoretical stances, which may impact how you collected and interpreted the data. For example, beliefs and values regarding overweight/obesity, guideline implementation or health promotion practice may impact your data collection/interpretation.

________________________________________________________________

________________________________________________________________

________________________________________________________________

________________________________________________________________

________________________________________________________________

Q6 Please reflect on whether believe these beliefs of values impacted data collection or interpretation (whether positively or negatively), and whether you used any strategies to mitigate any negative impacts

________________________________________________________________

________________________________________________________________

________________________________________________________________

________________________________________________________________

________________________________________________________________

Q7 Describe your professional background and how this may have impacted data collection and interpretation. For example, having a background in a health profession, qualitative research methods, etc.

________________________________________________________________

________________________________________________________________

________________________________________________________________

________________________________________________________________

________________________________________________________________

Q8 Please reflect on whether believe your professional background impacted data collection or interpretation (whether positively or negatively), and whether you used any strategies to mitigate any negative impacts

________________________________________________________________

________________________________________________________________

________________________________________________________________

________________________________________________________________

________________________________________________________________

Q9 Overall do you believe you had a significant influence on the data collected?

- Yes (1)
- No (2)

Display This Question:

If Overall do you believe you had a significant influence on the data collected? = Yes

Q10 If yes, how do you think you influenced the data collected?

________________________________________________________________

________________________________________________________________

________________________________________________________________

________________________________________________________________

________________________________________________________________

Q11 Did you encounter any issues with the online mode of data collection?

- Yes (1)
- No (2)

Display This Question:

If Did you encounter any issues with the online mode of data collection? = Yes

Q12 If yes, what were these issues?

________________________________________________________________

________________________________________________________________

________________________________________________________________

________________________________________________________________

________________________________________________________________

Q13 Do you feel the use of online data collection impacted data collection in any way?

- Yes (1)
- No (2)

Display This Question:

If Do you feel the use of online data collection impacted data collection in any way? = Yes

Q14 If yes, how was data collection impacted?

________________________________________________________________

________________________________________________________________

________________________________________________________________

________________________________________________________________

________________________________________________________________
