# Supplementary material for: The codesign of implementation strategies for children’s growth assessment guidelines in the dental setting
Source: Res Involv Engagem. 2022 May 16;8:19. doi: 10.1186/s40900-022-00356-8 (PMC9109434; doi:10.1186/s40900-022-00356-8)
Supplement: Supplementary file 4 — Additional file 4. Characteristics of the research team. [file 40900_2022_356_MOESM4_ESM.docx]

# Supplementary File 4: Characteristics of the research team

| **Researcher** | **Gender** | **Qualifications** | **Occupation** |
| --- | --- | --- | --- |
| Amy R. Villarosa | Female | BNutrDiet, MBiostat | PhD Candidate and Research Officer, Western Sydney University |
| Della Maneze | Female | MD, PhD | Multicultural Health Promotion Officer, South Western Sydney Local Health District and Adjunct Research Fellow, Western Sydney University |
| Lucie M. Ramjan | Female | BNurs (Hons), PhD | Associate Professor, School of Nursing and Midwifery, Western Sydney University |
| Ariana Kong | Female | BHSc (Hons), MPodMed | PhD Candidate and Research Officer, Western Sydney University |
| Ajesh George | Male | BDS, MPH, PhD | Professor, School of Nursing and Midwifery, Western Sydney University, and Director, Centre for Oral Health Outcomes and Research Translation |
