# Supplementary material for: The codesign of implementation strategies for children’s growth assessment guidelines in the dental setting
Source: Res Involv Engagem. 2022 May 16;8:19. doi: 10.1186/s40900-022-00356-8 (PMC9109434; doi:10.1186/s40900-022-00356-8)
Supplement: Supplementary file 5 — Additional file 5. Table of additional quotes. [file 40900_2022_356_MOESM5_ESM.docx]

# Supplementary File 5: Table of additional quotes

| **Theme** | **Subtheme** | **Quotes** |
| --- | --- | --- |
| Engaging families throughout their care journey | Setting expectations before the appointment | *Benefit of prior information about the guidelines*  “It's not a surprise, it's just happening, it's a standard procedure, there's some information there.” - Sarah  “We really have a clear idea of what exactly my son is going through” - Amita  “…if someone's coming for an appointment, telling them beforehand that this is going to happen so they don't feel like they've walked in the front door and you looked at their child and went, wow, that child looks overweight I'm going to do a height and weight on them.” - Sarah  *Benefit for teenagers and children with special needs*  “Yes, I have a teenage girl… You know what works the best for her? To have plans, to [know what’s] going to happen because when you change her plans.. she freaks out. That’s why I think that our strategy of setting it of expectations prior to the meeting is going to work with everything.” - Isabella  “kids with autism and stuff like that they need to be supported well ahead of time. If the situation were they’re not prepared… it’s just going to scare them to be honest.” - Joanne  *Strategies*  “…if someone's coming for an appointment, telling them beforehand that this is going to happen so they don't feel like they've walked in the front door and you looked at their child and went, wow, that child looks overweight I'm going to do a height and weight on them.” - Sarah  “It would be really simple to put that in the appointment letter” - Peter  “you’d have to get permission to get all that done and that can take time as well” - Natasha  “I think when we’re looking at sending information... People tend to prefer information to be emailed these days…” - Bianca |
|  | Personalised care during the appointment | *Benefit of personalised care*  “personalising it – being not as clinical or one-sided because the health care professionals often understand what they are saying but it is not exactly easy to understand as a normal civilian.” - Martina  “experiences that we've had, not being talked to by a health professional with one narrow message. The tailored messaging to the child, plus the parents I think increases engagement.” Mai  “Then he started to ask his own questions and be involved in how to get better. It was a completely different step for him. For me I wasn't the parent dragging him along anymore, he had a bit of ownership in it, and he was – I'd find him doing the exercises himself at home instead of me saying, okay, you've got to do these exercises now.” Sarah  *Dental staff agreement*  “it’s just explaining the full picture to them instead of just the end result.” - Penelope  “tailoring the advice and the [care] plans” - Bianca  “so many people are so individual what you say for one person is not going to work for somebody else.” - Leslie  “…parents want us to consider the patients themselves, the children… rather than just being told.” - Fatima  *Need for cultural sensitivity*  “I felt all the time that criticism, even in the way they were speaking to me, they spoke like … [by] raising their voice [I was going to] understand better and it was not the case. I could understand, but I couldn’t speak. That was my problem.” - Joanne  “in our clinic, we are very multicultural, so we need resources that will actually be able to educate them.” - Marie  “For us, we’re – yeah. We only have a Chinese restaurant, and that’s it.” - Jane |
|  | Continuity of care after the appointment | *Need for continuity of care*  “I knew where exactly we are headed in terms of the direction we should go with my son, what we should look out for.” – Amita  “some sort of clear follow through plan so that we could always go back to someone and get reassured, rather than it being this one event experience for the parent and child, and then they're move on to be referred to all these other programs, and it's never discussed again.” Mai  *Dental staff agreement*  “Being able to see that process of giving them the correct knowledge and being able to follow that through.. every.” - Bianca  *Need for updates from referral pathways*  “I would never really know if they’ve even done the Go4Fun [children’s healthy weight program] or anything like that unless I ask them the following time if they have or have not or whatever.” - Rosalie |
| Supporting staff to engage with the guidelines | Increasing intentions to engage in guidelines | *Positive attitudes/beliefs/confidence*  “it's actually [about] the overall health, the effect and the connection between the two [oral health and healthy weight]...” - Marie  “Sometimes they’re not seeing their GP for 18 months. That’s just dependant on whether they’re sick or due for a vaccine or something like that. In that time, sometimes they’re not getting their height, weight, and BMI assessed.” - Emma  *Negative attitudes/beliefs/confidence*  “it is basically an algorithm that’s pushed out of a computer that says that your child should be this.” - Leslie  “a specialist telling me that it is 90 per cent genetics, the black and white calculation to me is not the right the way to be doing the height and weight. Because it doesn’t take into account you have Chinese children or Chinese or Asian origin that will fall below a healthy weight. Yet you’ve got Tongan children at the same age that will be morbidly obese so there’s a lot more factors than just the height and weight.” - Leslie  “I understand why the government wants to do this but I do feel very cornered in giving people information that they most probably don’t want to know.” - Leslie  “I’m sorry but I’m not a trained physiotherapist, I’m not a dietitian, I’m a dental therapist. I deal with teeth. I’m not trained to do those things” - Leslie  *Patient acceptance*  “quite pleased about the fact that we’re doing it and we’re reinforcing that diet and their weight and their height is all related...” - Jane  “I find it that the ones that are a little bit hesitant are the teenage girls. That’s a different way to tackle but generally they still will do it, it’s just … they don’t want people to see how much they weigh.” - Natasha  “It may be hard to hear that your child is overweight or underweight or whatnot but I think in the long term you just need to do what’s best for your child.” - Lisa  *Need for information resources*  “pamphlets, because you need to educate them, so you have to give them some information.” - Lucy  “that tells parents why … the Ministry of Health has decided to do this.” - Leslie |
|  | Improving awareness and skills | *Need for staff training*  “If you expect anyone to do anything, you’ve got to give the knowledge and the training to do it otherwise they don’t know what they’re doing or why.” - Lucy  “we have to have a consistent message so perhaps the clinician … should have training on the consistency of what we're actually going present.” - Marie  *Need for refresher training*  “They have actually had training” - Penelope  “some of them are still not doing it correctly even after the training.” - Penelope  “we know about these programs, and we only have a brief understanding of how it's run. I think it's important that we, as clinicians, join with the Go4Fun groups for example, sit in and let them have a chat, explain to us exactly what they're doing. Then we can explain to the parents or the guardians how it's actually run, then we get a better relationship with these people that we're coming together with.” - Sofia  *Need for collaboration*  “A dietitian point of view is different from a dental therapist, oral health therapist point of view... I think it's a collaboration is what we need, and we need to be trained differently.” - Marie |
|  | Addressing environmental constraints | *Time restrictions*  “we haven’t got enough time and if we can train the assistants to be able to take the measurements, that’s a really big help.” - Jane  “when you are working with the therapist, well that’s what you do. We’ve all got certificates so we’re all able to do specific tasks if we’re trained to… I mean, I think [we] should be doing it.” - Bianca  *Scale location*  “located down the corridor from where the surgeries are.” - Rosalie  “the scales are actually in my clinic.” - Melissa  *Timing of assessment*  “Whether to do it at the first appointment or whether it is … more appropriate to make sure that we look after the pain rather than concentrate on height and weight at the beginning…” - Bianca  *Productivity evaluation*  “if … you’ve done three fillings in one side that’s more productive than what the dental health education is or the diet discussion in their eyes and in their view.” - Leslie  “So you could spend 20 minutes doing toothbrushing and discussion of diet and just an examination of a child’s mouth and their height and weight which we have to add into that half an hour. But the boss is saying, well that’s not enough work for half an hour. So how can the health department or Ministry of Health define it so that we’re getting the accolades for the work that we’re actually doing?” - Leslie  *Need for system to document longitudinal growth assessments*  “One of the things I’d like to be able to access much more simply… is the previous data. So, we could put that on a graph and show them the changes because some kids belong at the 80th percentile, some kids belong at the 20th percentile. But as long as we can show them that they’re steadily being the same… I would like to be able to compare over visits much more easily than we can.” - Peter |
